# Terminologia Medica Polyglotta

**Published:** 1890-09

**Authors:** 


TERMINOLOGIA MEDICA POLYGLOTTA. A Concise International Dictionary of Medical Terms. Compiled by THEODORE MAXWELL, M. D., Camb., B. Sc., Lond., F. R. C. S., Edin.; with the Assistance of Nine Collaborators. In Latin, English, French, German, Italian, and Spanish. 8vo; pp. xvi.–459. London: J. & A. Churchill. Paris: G. Masson. Philadelphia: P. Blakiston, Son & Co. 1890.

This work is unique in medical literature, and represents an enormous amount of painstaking work by the author. Its declared purpose is "to assist medical men of various nationalties to read the medical literature of other countries."

It embraces about 25,000 words, probably a sufficient number for the needs of those who will seek the assistance of its pages, and French has been selected as the key-language. Its arrangement is simple and convenient, and it must become an indispensable part of the library to those who would perfect themselves in medical terminology; hence teachers, writers, and scholars will appreciate and possess it. The letterpress, paper and binding are beautiful.

